# Supplementary material for: Taxonomic revision of the tarantula genus Aphonopelma Pocock, 1901 (Araneae, Mygalomorphae, Theraphosidae) within the United States
Source: Zookeys. 2016 Feb 4;(560):1–340. doi: 10.3897/zookeys.560.6264 (PMC4768370; doi:10.3897/zookeys.560.6264)
Supplement: Supplementary material 2 — Compressed files (.zip) containing Aphonopelma boxplot and PCA morphospace data. [file zookeys-560-001-s002.zip › Supp2b.zip!/Appendix 2B/Boxplot_morphospace/Aphonopelma_boxplots_females/Pygmy/A1A3_ratio_females.pdf]

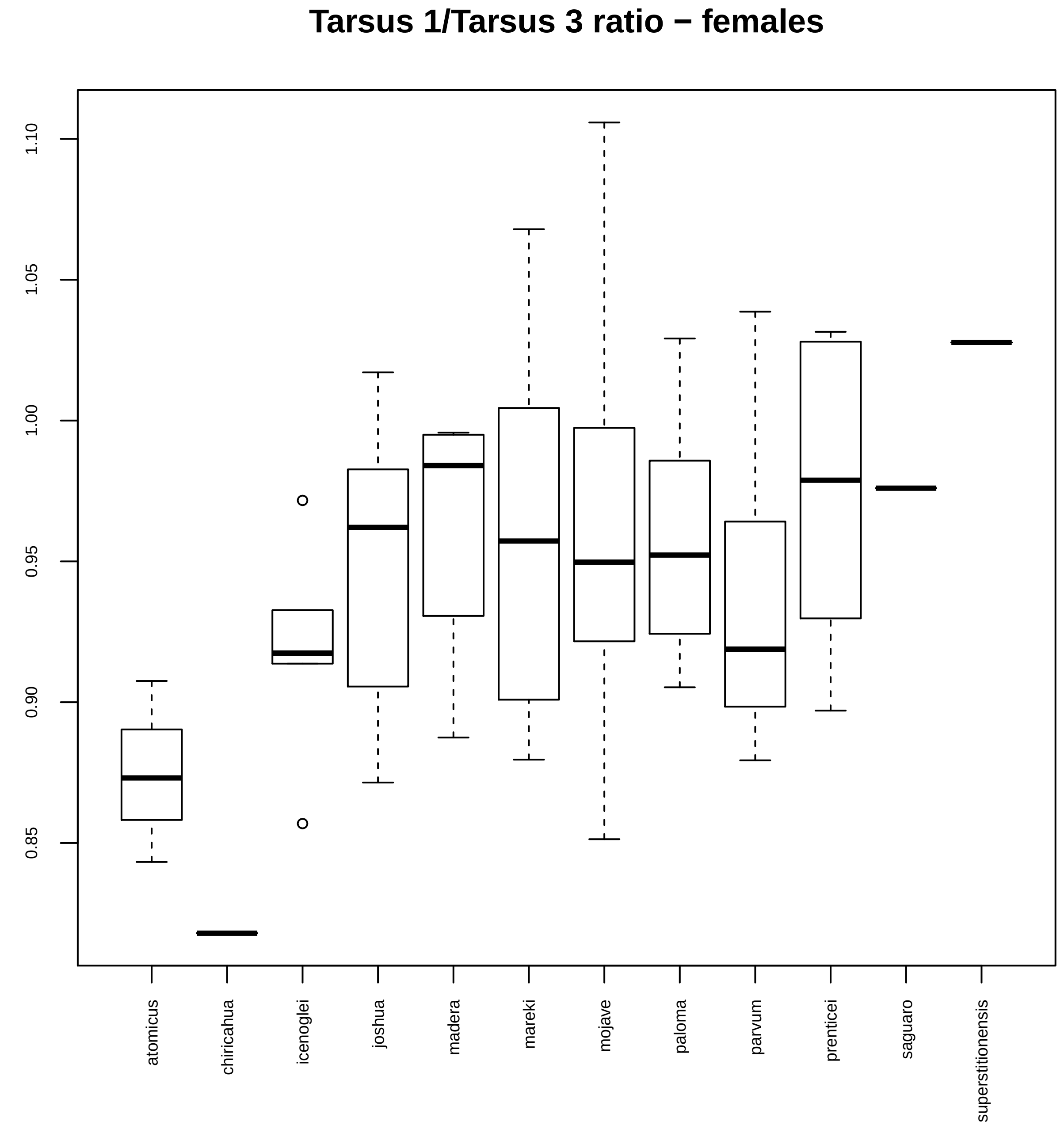

Tarsus 1/Tarsus 3 ratio − females
0.85
0.90
0.95
1.00
1.05
1.10
atomicus
chiricahua
icenoglei
joshua
madera
mareki
mojave
paloma
parvum
prenticei
saguaro
superstitionensis
